# 2,4-Dimethyl-*N*-(2-methyl­phen­yl)benzene­sulfonamide

**DOI:** 10.1107/S1600536809049563

**Published:** 2009-11-25

**Authors:** P. G. Nirmala, B. Thimme Gowda, Sabine Foro, Hartmut Fuess

**Affiliations:** a Department of Chemistry, Mangalore University, Mangalagangotri 574 199, Mangalore, India; b Institute of Materials Science, Darmstadt University of Technology, Petersenstrasse 23, D-64287 Darmstadt, Germany

## Abstract

In the title compound, C_15_H_17_NO_2_S, the mol­ecule is bent at the S atom with a C—SO_2_—NH—C torsion angle of 71.6 (1)°. The two benzene rings are tilted by 47.0 (1)° relative to each other. The crystal structure features inversion-related dimers linked by pairs of N—H⋯O hydrogen bonds.

## Related literature

For the preparation of the title compound, see: Savitha & Gowda (2006 ▶). For related structures, see: Gelbrich *et al.* (2007 ▶); Gowda *et al.* (2008 ▶; 2009**a* ▶,b* ▶); Perlovich *et al.* (2006 ▶).
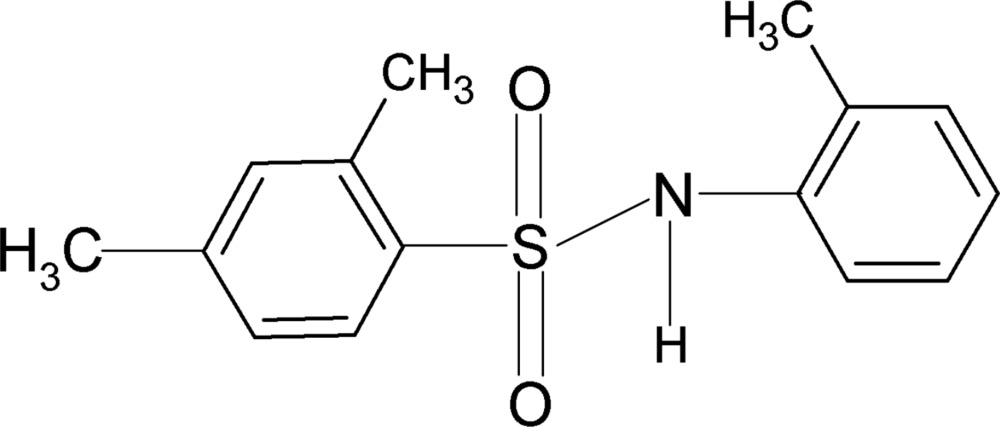


## Experimental

### 

#### Crystal data

C_15_H_17_NO_2_S
*M* *_r_* = 275.36
Triclinic, 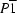

*a* = 8.1789 (8) Å
*b* = 8.2659 (9) Å
*c* = 11.005 (1) Å
α = 96.249 (9)°
β = 96.078 (9)°
γ = 106.782 (9)°
*V* = 700.68 (12) Å^3^
*Z* = 2
Mo *K*α radiation
μ = 0.23 mm^−1^
*T* = 299 K
0.48 × 0.26 × 0.12 mm

#### Data collection

Oxford Diffraction Xcalibur diffractometer with a Sapphire CCD detector
Absorption correction: multi-scan (*CrysAlis RED*; Oxford Diffraction, 2009 ▶) *T*_min_ = 0.898, *T*_max_ = 0.973
4894 measured reflections
2862 independent reflections
2446 reflections with *I* > 2σ(*I*)
*R*_int_ = 0.010

#### Refinement

*R*[*F* ^2^ > 2σ(*F* ^2^)] = 0.039
*wR*(*F* ^2^) = 0.116
*S* = 1.05
2862 reflections
178 parameters
1 restraint
H atoms treated by a mixture of independent and constrained refinement
Δρ_max_ = 0.39 e Å^−3^
Δρ_min_ = −0.24 e Å^−3^

### 

Data collection: *CrysAlis CCD* (Oxford Diffraction, 2009 ▶); cell refinement: *CrysAlis RED* (Oxford Diffraction, 2009 ▶); data reduction: *CrysAlis RED*; program(s) used to solve structure: *SHELXS97* (Sheldrick, 2008 ▶); program(s) used to refine structure: *SHELXL97* (Sheldrick, 2008 ▶); molecular graphics: *PLATON* (Spek, 2009 ▶); software used to prepare material for publication: *SHELXL97*.

## Supplementary Material

Crystal structure: contains datablocks I, global. DOI: 10.1107/S1600536809049563/ci2971sup1.cif

Structure factors: contains datablocks I. DOI: 10.1107/S1600536809049563/ci2971Isup2.hkl

Additional supplementary materials:  crystallographic information; 3D view; checkCIF report

## Figures and Tables

**Table 1:** Hydrogen-bond geometry (Å, °)

*D*—H⋯*A*	*D*—H	H⋯*A*	*D*⋯*A*	*D*—H⋯*A*
N1—H1*N*⋯O1^i^	0.83 (2)	2.19 (2)	3.002 (2)	165 (2)

Symmetry code: (i) 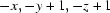.

**Fig. 1.**
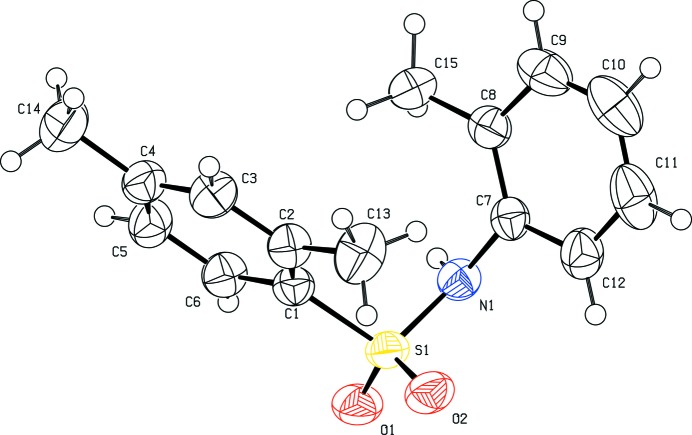
Molecular structure of (I), showing the atom labelling scheme. Displacement ellipsoids are drawn at the 50% probability level.

**Fig. 2.**
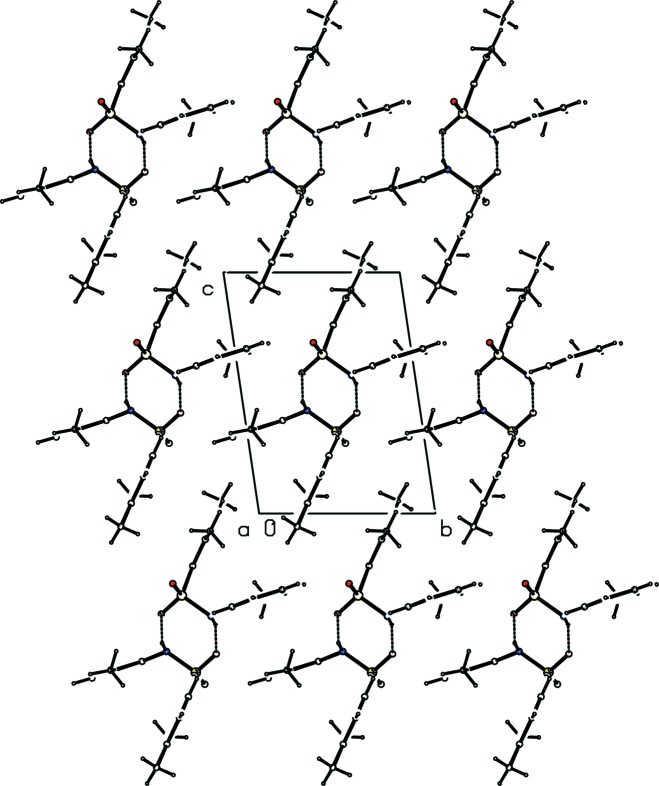
Molecular packing of (I) with hydrogen bonding shown as dashed lines.

## supplementary crystallographic information

### Comment

As part of a study of the substituent effects on the structures of *N*-(aryl)-arylsulfonamides (Gowda *et al.*, 2008; 2009*a,b*), in the present work, the structure of 2,4-dimethyl-*N*-(2-methylphenyl)benzenesulfonamide (I) has been determined (Fig. 1). The molecule is bent at the S atom with the C—SO_2_—NH—C torsion angle of 71.6 (1)°, compared with the values of 73.0 (2)° in 4-chloro-2-methyl-*N*-(2-methylphenyl)benzenesulfonamide (II) (Gowda *et al.* 2009*b*), -46.1 (3)° (molecule 1) and 47.7 (3)° (molecule 2) in the two molecules of 2,4-dimethyl-*N*-(phenyl)benzenesulfonamide (III) (Gowda *et al.* 2009*a*) and 72.0 (2)° in *N*-(2-methylphenyl)benzenesulfonamide (IV) (Gowda *et al.*, 2008). The two benzene rings in (I) are tilted relative to each other by 47.0 (1)°, compared to the values of 45.8 (1)° in (II), 67.5 (1)° in molecule 1 and 72.9 (1)° in molecule 2 of (III), and 61.5 (1)° in (IV). The other bond parameters in (I) are similar to those observed in (II), (III), (IV) and other aryl sulfonamides(Perlovich *et al.*, 2006; Gelbrich *et al.*, 2007). The crystal packing of molecules in (I) is characterized by N—H···O hydrogen bonds (Table 1, Fig.2).

### Experimental

A solution of 1,3-xylene (1,3-dimethylbenzene) (10 ml) in chloroform (40 ml) was treated dropwise with chlorosulfonic acid (25 ml) at 0 ° C. After the initial evolution of hydrogen chloride subsided, the reaction mixture was brought to room temperature and poured into crushed ice in a beaker. The chloroform layer was separated, washed with cold water and allowed to evaporate slowly. The residual 2,4-dimethylbenzenesulfonylchloride was treated with *o*-toluidine in the stoichiometric ratio and boiled for ten minutes. The reaction mixture was then cooled to room temperature and added to ice cold water (100 ml). The resultant solid 2,4-dimethyl-*N*-(2-methylphenyl)benzenesulfonamide was filtered under suction and washed thoroughly with cold water. It was then recrystallized to constant melting point from dilute ethanol. The purity of the compound was checked and characterized by recording its infrared and NMR spectra (Savitha & Gowda, 2006). The prism like colourless single crystals used in X-ray diffraction studies were grown in ethanolic solution by a slow evaporation at room temperature.

### Refinement

The H atom of the NH group was located in a difference map and its position was refined with the N-H distance restrained to 0.86 (2) Å. The other H atoms were positioned with idealized geometry using a riding model [C-H = 0.93–0.96 Å]. All H atoms were refined with isotropic displacement parameters (set to 1.2 times of the *U*_eq_ of the parent atom).

### Crystal data

**Table:**

C_15_H_17_NO_2_S	*Z* = 2
*M**_r_* = 275.36	*F*(000) = 292
Triclinic, *P*1	*D*_x_ = 1.305 Mg m^−^^3^
Hall symbol: -P 1	Mo *K*α radiation, λ = 0.71073 Å
*a* = 8.1789 (8) Å	Cell parameters from 2978 reflections
*b* = 8.2659 (9) Å	θ = 3.0–27.9°
*c* = 11.005 (1) Å	µ = 0.23 mm^−^^1^
α = 96.249 (9)°	*T* = 299 K
β = 96.078 (9)°	Prism, colourless
γ = 106.782 (9)°	0.48 × 0.26 × 0.12 mm
*V* = 700.68 (12) Å^3^	

### Data collection

**Table:**

Oxford Diffraction Xcalibur diffractometer with a Sapphire CCD detector	2862 independent reflections
Radiation source: fine-focus sealed tube	2446 reflections with *I* > 2σ(*I*)
graphite	*R*_int_ = 0.010
Rotation method data acquisition using ω and φ scans	θ_max_ = 26.4°, θ_min_ = 3.0°
Absorption correction: multi-scan (*CrysAlis RED*; Oxford Diffraction, 2009)	*h* = −10→10
*T*_min_ = 0.898, *T*_max_ = 0.973	*k* = −10→7
4894 measured reflections	*l* = −13→13

### Refinement

**Table:**

Refinement on *F*^2^	Primary atom site location: structure-invariant direct methods
Least-squares matrix: full	Secondary atom site location: difference Fourier map
*R*[*F*^2^ > 2σ(*F*^2^)] = 0.039	Hydrogen site location: inferred from neighbouring sites
*wR*(*F*^2^) = 0.116	H atoms treated by a mixture of independent and constrained refinement
*S* = 1.05	*w* = 1/[σ^2^(*F*_o_^2^) + (0.064*P*)^2^ + 0.2128*P*] where *P* = (*F*_o_^2^ + 2*F*_c_^2^)/3
2862 reflections	(Δ/σ)_max_ = 0.001
178 parameters	Δρ_max_ = 0.39 e Å^−^^3^
1 restraint	Δρ_min_ = −0.24 e Å^−^^3^

### Special details

**Table:**

Geometry. All e.s.d.'s (except the e.s.d. in the dihedral angle between two l.s. planes) are estimated using the full covariance matrix. The cell e.s.d.'s are taken into account individually in the estimation of e.s.d.'s in distances, angles and torsion angles; correlations between e.s.d.'s in cell parameters are only used when they are defined by crystal symmetry. An approximate (isotropic) treatment of cell e.s.d.'s is used for estimating e.s.d.'s involving l.s. planes.
Refinement. Refinement of *F*^2^ against ALL reflections. The weighted *R*-factor *wR* and goodness of fit *S* are based on *F*^2^, conventional *R*-factors *R* are based on *F*, with *F* set to zero for negative *F*^2^. The threshold expression of *F*^2^ > σ(*F*^2^) is used only for calculating *R*-factors(gt) *etc*. and is not relevant to the choice of reflections for refinement. *R*-factors based on *F*^2^ are statistically about twice as large as those based on *F*, and *R*- factors based on ALL data will be even larger.

### Fractional atomic coordinates and isotropic or equivalent isotropic displacement parameters (Å^2^)

**Table:**

	*x*	*y*	*z*	*U*_iso_*/*U*_eq_	
C1	0.1125 (2)	0.5794 (2)	0.78239 (16)	0.0390 (4)	
C2	0.2116 (2)	0.6728 (2)	0.89249 (16)	0.0440 (4)	
C3	0.1243 (3)	0.7397 (3)	0.97740 (17)	0.0496 (4)	
H3	0.1876	0.8030	1.0513	0.060*	
C4	−0.0514 (3)	0.7168 (2)	0.95762 (18)	0.0480 (4)	
C5	−0.1451 (2)	0.6234 (3)	0.84761 (18)	0.0505 (4)	
H5	−0.2633	0.6063	0.8322	0.061*	
C6	−0.0645 (2)	0.5551 (2)	0.76046 (17)	0.0458 (4)	
H6	−0.1287	0.4925	0.6866	0.055*	
C7	0.4102 (2)	0.7886 (2)	0.61387 (14)	0.0380 (4)	
C8	0.3900 (2)	0.9481 (2)	0.64940 (15)	0.0432 (4)	
C9	0.5397 (3)	1.0860 (3)	0.68291 (19)	0.0599 (5)	
H9	0.5304	1.1944	0.7053	0.072*	
C10	0.7000 (3)	1.0659 (3)	0.6837 (2)	0.0693 (7)	
H10	0.7978	1.1600	0.7075	0.083*	
C11	0.7175 (3)	0.9075 (3)	0.6495 (2)	0.0691 (7)	
H11	0.8268	0.8942	0.6511	0.083*	
C12	0.5722 (2)	0.7676 (3)	0.61251 (19)	0.0525 (5)	
H12	0.5831	0.6607	0.5870	0.063*	
C13	0.4036 (3)	0.7076 (3)	0.9262 (2)	0.0642 (6)	
H13A	0.4627	0.7664	0.8654	0.077*	
H13B	0.4287	0.6014	0.9290	0.077*	
H13C	0.4416	0.7771	1.0057	0.077*	
C14	−0.1363 (3)	0.7946 (3)	1.0542 (2)	0.0660 (6)	
H14A	−0.0497	0.8623	1.1206	0.079*	
H14B	−0.2169	0.7053	1.0855	0.079*	
H14C	−0.1960	0.8657	1.0176	0.079*	
C15	0.2154 (3)	0.9733 (2)	0.6503 (2)	0.0583 (5)	
H15A	0.1600	0.9167	0.7135	0.070*	
H15B	0.1462	0.9261	0.5714	0.070*	
H15C	0.2283	1.0932	0.6667	0.070*	
N1	0.26247 (19)	0.64126 (19)	0.57272 (13)	0.0409 (3)	
H1N	0.175 (2)	0.659 (3)	0.5378 (18)	0.049*	
O1	0.06289 (17)	0.36111 (16)	0.58290 (13)	0.0527 (3)	
O2	0.35102 (17)	0.45144 (17)	0.70879 (13)	0.0524 (3)	
S1	0.20156 (5)	0.49271 (5)	0.65960 (4)	0.04048 (16)	

### Atomic displacement parameters (Å^2^)

**Table:**

	*U*^11^	*U*^22^	*U*^33^	*U*^12^	*U*^13^	*U*^23^
C1	0.0394 (9)	0.0368 (8)	0.0420 (9)	0.0129 (7)	0.0056 (7)	0.0065 (7)
C2	0.0413 (9)	0.0472 (9)	0.0434 (9)	0.0139 (8)	0.0035 (7)	0.0074 (7)
C3	0.0527 (11)	0.0531 (11)	0.0422 (9)	0.0177 (9)	0.0034 (8)	0.0025 (8)
C4	0.0538 (11)	0.0481 (10)	0.0500 (10)	0.0232 (9)	0.0146 (8)	0.0127 (8)
C5	0.0404 (9)	0.0584 (11)	0.0571 (11)	0.0200 (9)	0.0087 (8)	0.0123 (9)
C6	0.0412 (9)	0.0480 (10)	0.0473 (10)	0.0143 (8)	0.0020 (7)	0.0051 (8)
C7	0.0374 (8)	0.0422 (9)	0.0328 (8)	0.0095 (7)	0.0055 (6)	0.0050 (6)
C8	0.0522 (10)	0.0408 (9)	0.0344 (8)	0.0100 (8)	0.0084 (7)	0.0046 (7)
C9	0.0737 (14)	0.0435 (10)	0.0498 (11)	0.0012 (10)	0.0025 (10)	0.0043 (8)
C10	0.0565 (13)	0.0683 (14)	0.0624 (13)	−0.0109 (11)	−0.0078 (10)	0.0199 (11)
C11	0.0368 (10)	0.0928 (18)	0.0765 (15)	0.0099 (11)	0.0044 (10)	0.0380 (13)
C12	0.0426 (10)	0.0627 (12)	0.0579 (11)	0.0201 (9)	0.0117 (8)	0.0176 (9)
C13	0.0440 (11)	0.0873 (16)	0.0544 (12)	0.0194 (11)	−0.0049 (9)	−0.0059 (11)
C14	0.0683 (14)	0.0747 (15)	0.0644 (13)	0.0333 (12)	0.0219 (11)	0.0065 (11)
C15	0.0692 (13)	0.0461 (11)	0.0683 (13)	0.0261 (10)	0.0246 (11)	0.0074 (9)
N1	0.0391 (8)	0.0405 (8)	0.0409 (8)	0.0117 (6)	0.0020 (6)	0.0006 (6)
O1	0.0516 (7)	0.0371 (6)	0.0622 (8)	0.0085 (6)	0.0031 (6)	−0.0050 (6)
O2	0.0499 (7)	0.0472 (7)	0.0660 (8)	0.0246 (6)	0.0075 (6)	0.0078 (6)
S1	0.0399 (2)	0.0329 (2)	0.0477 (3)	0.01217 (17)	0.00452 (18)	0.00091 (17)

### Geometric parameters (Å, °)

**Table:**

C1—C6	1.394 (2)	C10—C11	1.375 (4)
C1—C2	1.396 (2)	C10—H10	0.93
C1—S1	1.7799 (17)	C11—C12	1.384 (3)
C2—C3	1.394 (3)	C11—H11	0.93
C2—C13	1.510 (2)	C12—H12	0.93
C3—C4	1.385 (3)	C13—H13A	0.96
C3—H3	0.93	C13—H13B	0.96
C4—C5	1.381 (3)	C13—H13C	0.96
C4—C14	1.513 (3)	C14—H14A	0.96
C5—C6	1.380 (3)	C14—H14B	0.96
C5—H5	0.93	C14—H14C	0.96
C6—H6	0.93	C15—H15A	0.96
C7—C12	1.386 (2)	C15—H15B	0.96
C7—C8	1.395 (2)	C15—H15C	0.96
C7—N1	1.436 (2)	N1—S1	1.6350 (16)
C8—C9	1.393 (3)	N1—H1N	0.832 (15)
C8—C15	1.503 (3)	O1—S1	1.4377 (13)
C9—C10	1.368 (3)	O2—S1	1.4299 (13)
C9—H9	0.93		
			
C6—C1—C2	120.83 (16)	C12—C11—H11	120.1
C6—C1—S1	115.77 (13)	C11—C12—C7	119.3 (2)
C2—C1—S1	123.34 (13)	C11—C12—H12	120.3
C3—C2—C1	116.59 (16)	C7—C12—H12	120.3
C3—C2—C13	117.89 (17)	C2—C13—H13A	109.5
C1—C2—C13	125.51 (17)	C2—C13—H13B	109.5
C4—C3—C2	123.53 (18)	H13A—C13—H13B	109.5
C4—C3—H3	118.2	C2—C13—H13C	109.5
C2—C3—H3	118.2	H13A—C13—H13C	109.5
C5—C4—C3	118.18 (17)	H13B—C13—H13C	109.5
C5—C4—C14	121.52 (18)	C4—C14—H14A	109.5
C3—C4—C14	120.30 (19)	C4—C14—H14B	109.5
C6—C5—C4	120.45 (17)	H14A—C14—H14B	109.5
C6—C5—H5	119.8	C4—C14—H14C	109.5
C4—C5—H5	119.8	H14A—C14—H14C	109.5
C5—C6—C1	120.42 (17)	H14B—C14—H14C	109.5
C5—C6—H6	119.8	C8—C15—H15A	109.5
C1—C6—H6	119.8	C8—C15—H15B	109.5
C12—C7—C8	121.51 (17)	H15A—C15—H15B	109.5
C12—C7—N1	117.73 (16)	C8—C15—H15C	109.5
C8—C7—N1	120.71 (15)	H15A—C15—H15C	109.5
C9—C8—C7	117.21 (18)	H15B—C15—H15C	109.5
C9—C8—C15	120.60 (18)	C7—N1—S1	121.46 (11)
C7—C8—C15	122.19 (16)	C7—N1—H1N	116.5 (14)
C10—C9—C8	121.6 (2)	S1—N1—H1N	108.7 (15)
C10—C9—H9	119.2	O2—S1—O1	118.89 (8)
C8—C9—H9	119.2	O2—S1—N1	108.05 (8)
C9—C10—C11	120.4 (2)	O1—S1—N1	104.94 (8)
C9—C10—H10	119.8	O2—S1—C1	109.57 (8)
C11—C10—H10	119.8	O1—S1—C1	107.40 (8)
C10—C11—C12	119.9 (2)	N1—S1—C1	107.42 (8)
C10—C11—H11	120.1		
			
C6—C1—C2—C3	−0.2 (3)	C15—C8—C9—C10	179.47 (19)
S1—C1—C2—C3	176.86 (13)	C8—C9—C10—C11	0.9 (3)
C6—C1—C2—C13	−179.88 (19)	C9—C10—C11—C12	0.8 (4)
S1—C1—C2—C13	−2.8 (3)	C10—C11—C12—C7	−1.8 (3)
C1—C2—C3—C4	0.4 (3)	C8—C7—C12—C11	1.2 (3)
C13—C2—C3—C4	−179.95 (19)	N1—C7—C12—C11	178.85 (17)
C2—C3—C4—C5	−0.3 (3)	C12—C7—N1—S1	75.97 (18)
C2—C3—C4—C14	−179.61 (19)	C8—C7—N1—S1	−106.39 (17)
C3—C4—C5—C6	0.0 (3)	C7—N1—S1—O2	−46.56 (15)
C14—C4—C5—C6	179.35 (18)	C7—N1—S1—O1	−174.35 (12)
C4—C5—C6—C1	0.1 (3)	C7—N1—S1—C1	71.57 (14)
C2—C1—C6—C5	0.0 (3)	C6—C1—S1—O2	−152.26 (13)
S1—C1—C6—C5	−177.30 (14)	C2—C1—S1—O2	30.51 (17)
C12—C7—C8—C9	0.3 (3)	C6—C1—S1—O1	−21.81 (16)
N1—C7—C8—C9	−177.20 (15)	C2—C1—S1—O1	160.95 (14)
C12—C7—C8—C15	179.46 (17)	C6—C1—S1—N1	90.60 (14)
N1—C7—C8—C15	1.9 (3)	C2—C1—S1—N1	−86.63 (16)
C7—C8—C9—C10	−1.4 (3)		

### Hydrogen-bond geometry (Å, °)

**Table:**

*D*—H···*A*	*D*—H	H···*A*	*D*···*A*	*D*—H···*A*
N1—H1N···O1^i^	0.83 (2)	2.19 (2)	3.002 (2)	165 (2)

Symmetry codes: (i) −*x*, −*y*+1, −*z*+1.
